# Safety and Patient Acceptability of Stellate Ganglion Blockade as a Treatment Adjunct for Combat-Related Post-Traumatic Stress Disorder: A Quality Assurance Initiative

**DOI:** 10.7759/cureus.320

**Published:** 2015-09-10

**Authors:** Brian McLean

**Affiliations:** 1 Tripler Army Medical Center

**Keywords:** post traumatic stress disorders, stellate ganglion block, sgb, ptsd, sympathetic nervous system, sympathectomy, qa, combat military veterans, military, anxiety

## Abstract

OBJECTIVE: To perform a quality assurance and performance improvement project through review of our single center data on the safety and patient acceptability of the stellate ganglion blockade (SGB) procedure for the relief of symptoms related to chronic post-traumatic stress disorder.

BACKGROUND: Our interventional pain management service has been offering trials of SGB therapy to assist with the management of the sympathetically mediated anxiety and hyperarousal symptoms of severe and treatment-refractory combat-related PTSD. There have been multiple case series in the literature describing the potential impact of this procedure for PTSD symptom management as well as the safety of image-guided procedures. We wished to ensure that we were performing this procedure safely and that patients were tolerating and accepting of this adjunctive treatment option.

METHODS: We conducted a review of our quality assurance and performance improvement data over the past 18 months during which we performed 250 stellate ganglion blocks for the management of PTSD symptoms to detect any potential complications or unanticipated side effects.  We also analyzed responses from an anonymous patient de-identified survey collected regarding the comfort and satisfaction associated with the procedure.

RESULTS: We did not identify any immediate post-procedural complications or delayed complications from any of the 250 procedures performed from November 2013 to April 2015. Of the 110 surveys that were returned and tabulated, 100% of the patients surveyed were overall satisfied with our process and with the procedure, 100% said they would recommend the procedure to a friend, and 95% stated that they would be willing to undergo as many repeat procedures as necessary based on little discomfort and tolerable side effects.

CONCLUSION: Our quality assurance assessment suggests that in our center the SGB procedure for PTSD is a safe, well-tolerated, and acceptable treatment adjunct in the management of severe symptoms associated with chronic treatment-refractory PTSD. Patient satisfaction responses are strongly suggestive of high therapeutic value, and further studies are indicated to determine the effectiveness, duration of action, and optimal treatment regimen.

## Introduction

Disclaimer: The views expressed in this abstract/manuscript are those of the author and do not reflect the official policy or position of the Department of the Army, Department of Defense, or the US Government.

Post-traumatic stress disorder (PTSD) is a devastating condition that contributes to significant functional impairment in affected individuals. Rates of combat-related PTSD in U.S. veterans are estimated to range between 2 - 17% with a lifetime prevalence of 3 - 31% [1]. A few therapies have shown benefit in the treatment of PTSD, such as pharmacotherapies (selective serotonin reuptake inhibitors) and psychotherapies (cognitive behavioral therapy and exposure-based therapies) [2]. However, many patients struggle for years with treatment-resistant symptoms and functional disability from combat-related PTSD, which has led to a search for alternative treatments.

Based on the author’s experience and previous case series regarding the potential benefit of stellate ganglion blockade (SGB) for the management of PTSD symptoms, we have been offering the procedure at our interventional pain management center for the past five years. The SGB is a selective block of the cervical sympathetic chain designed to reduce autonomic reactivity. It has long been effectively used in the treatment of pain conditions, such as complex regional pain syndrome, atypical facial pain, and sympathetically-mediated pain of the upper extremity. More recently, SGB has demonstrated an effective treatment of other conditions, including hot flashes [3]. Previously published case reports have demonstrated promising effects of SGB in decreasing some of the severe symptoms related to PTSD [4-9].

In light of the fact that this is a novel use of SGB in a vulnerable patient population, we sought to undertake a quality assurance and performance improvement initiative to assure that we were having safe outcomes and that our patients were tolerating the procedure; we felt that it was an acceptable treatment option.

## Materials and methods

Patients were provided with written and verbal information about the availability of the procedure as a method to help manage sympathetic arousal symptoms. In addition, risks and potential benefits of SGB were discussed. If requested, the patient was scheduled for the procedure, and informed consent was obtained. After informed consent, an intravenous line was started with a 22G IV in the patient’s upper extremity. The patient was positioned comfortably in the supine position with the head rotated to the contralateral side and prepped and draped in the sterile fashion. Radiographic confirmation of the right C6 transverse process was obtained using c-arm fluoroscopy. The skin was anesthetized with 0.5 cc of 2% lidocaine. Using a lateral oblique approach, a 25-gauge Quincke needle was passed under fluoroscopic guidance until it contacted the anterior lateral body of the C6 vertebra and then was pulled back approximately 0.5 mm. Appropriate needle position was then confirmed by injection of 2 cc of iohexol (180 mg/mL) radio-opaque dye to monitor its spread. After negative aspiration, a 2 ml test dose of 2% lidocaine was slowly injected, and after negative test dose, an additional 5 ml of 0.5% ropivacaine was slowly injected to produce a sympathetic block. After the needle was withdrawn and a Band-Aid applied, the patient was monitored for a minimum of 30 minutes and observed for complications and appropriate cervical sympathetic blockade as evidenced by facial anhidrosis and Horner’s syndrome symptoms (namely, ptosis, miosis, and scleral injection). For this quality assurance project, we did not collect data as to the percentage of a success blockade or clinical response to the block at the time of the survey.

Patients were given post-procedure instructions that included anticipated symptoms, potential side effects, and a call back number to report side effects, ask questions, or to schedule a repeat block should they have a prolonged and positive response to the first block. During recovery, the patients were also given a de-identified quality assurance and performance improvement survey approved by hospital command (Deputy Chief of Clinical Services) and our IRB Committee (Tripler Army Medical Center Department of Clinical Investigation IRB who approved this study as a QA/QI project) (Appendix 1). All patients who underwent SGB after project approval were given a survey in the post-procedure recovery area and asked to turn it into a folder at the time of discharge from the clinic.

As part of our normal process improvement and ongoing professional performance evaluation, we collect data on all procedures performed in our center to track any complications or unintended outcomes. This includes the collection of adverse outcome forms at the time of the procedure as well as any time after the procedure. As part of our annual performance review, we evaluate all procedures performed to compare our outcomes with national benchmarks and report our findings to the hospital invasive procedure review committee. We used these data to identify if any SGB procedure resulted in a complication or unanticipated side effect. The performance indicators and complications that we track are listed in Appendix 2.

## Results

From November 2013 to April 2015, we performed 250 SGB procedures specifically for the management of PTSD symptoms. We did not identify or record any immediate or delayed complications or unanticipated side effects from these procedures.

### Quality assurance survey results

After hospital command and IRB approval of our quality assurance and performance improvement project in October 2014, we began handing out our survey in the immediate post-procedure recovery area. Of the survey respondents, 51.8% had undergone one procedure, 24.6% had undergone two procedures, and 23.6% had undergone three or more procedures.

The procedure was well tolerated by all patients with no significant complications or side effects.  The results of the survey are detailed below in Table 1.

**Table 1:** Survey Results

Question	Number of Respondents	Percentage of Responses
What treatment number was this?		
1	57	51.8%
2	27	24.5%
3	13	11.8%
>=4	13	11.8%
How uncomfortable was the procedure for you?		
No discomfort	43	39.4%
Mild discomfort	54	49.5%
Moderate discomfort	11	10.1%
Severe discomfort	1	0.9%
How would you rate any side effects you had from this procedures compared to other treatments you have had?		
The same	44	40.0%
Less	55	50.0%
More	11	10.0%
Would you be willing to have this procedure again?		
Yes	110	100.0%
No	0	0.0%
Would you recommend the procedure to a friend?		
Yes	110	100.0%
No	0	0.0%
How many procedures would you be willing to have assuming it was an effective treatment?		
One more	0	0.0%
2-3 more	6	5.5%
As many as it takes	104	94.5%
How satisfied are you with the process and the education you received prior to and after the procedure		
Satisfied	108	98.2%
Neutral	2	1.8%
Dissatisfied	0	0.0%
Overall satisfied?		
Yes	110	100.0%
No	0	0.0%

## Discussion

The SGB procedure has inherent risks of procedural complications that include a misplaced needle puncturing adjacent structures resulting in hematoma, bleeding, and nerve, vessel, or other tissue injury as well as inadvertent spread of local anesthetic to surrounding structures and accidental intravascular injection. There is a very small risk of infection, which can be minimized by adherence to strict sterile procedure. Significant complications of the procedure are thought to be very rare but are not well defined by recent literature. In 1992, Wulf and Maier published a review that reported the incidence of severe complications as 1.7 in 1,000; however, this review was prior to the procedure being performed with image guidance, and at the time, a blind technique was commonly used [10]. We anticipate an exceedingly small rate of major complications using fluoroscopic or ultrasound guidance. Placement of an intravenous line, vital sign monitoring, and performance of the procedure in a non-sedated patient likely further decreases the complication risk. In our center, only board certified pain physicians using fluoroscopic guidance performed SGBs. Although we did not detect any significant complications during our fluoroscopically-guided procedures, there is discussion and suggestion in the literature that ultrasound guidance may provide an enhanced level of safety. Whereas fluoroscopic guidance can detect intravascular injection via the application of contrast media prior to injection of a local anesthetic, ultrasound guidance may prevent intravascular injection via real-time needle placement and injection. We feel that the risk for either image-guided procedure is low; however, we will continue to work to ensure that we are performing the safest possible procedure for our patients. Since the time of this report, we have started to transition to ultrasound guidance.

As part of our pre-procedure counseling, we describe to our patients what to expect during the procedure. Most commonly, patients undergoing SGB may experience mild discomfort during and after the procedure, a “full” sensation in the throat, and symptoms consistent with Horner’s syndrome (ptosis, anhidrosis, miosis, and scleral injection) consistent with a successful SGB. These mild symptoms are short-lived and in almost all cases resolve within hours. For our analysis, we do not consider these typical symptoms to be complications or side effects. However, in answering the survey question about side effects from the treatment, it is possible that patients will consider these symptoms to be side effects of the procedure. Despite this, the majority rated the side effects as the same or less compared to conventional PTSD treatments, speaking to the unfortunate side effects of the psychoactive medications currently used for the amelioration of PTSD symptoms.

The fact that 100% of our patients stated that they were overall satisfied with SGB and would be willing to recommend the procedure to a friend, in addition to a willingness to undergo repeated procedures as necessary, indicates to us that the procedure is a well-tolerated and accepted adjunct treatment option for patients with combat-related PTSD.

Our quality assurance project was not designed to capture or evaluate outcomes of SGB for PTSD, and therefore, further studies are indicated to determine the effectiveness, duration of action, and optimal treatment regimen. Surveys were given to all patients immediately following the procedure and the patient completed and turned in the survey in the immediate post-procedure recovery area. To maintain anonymity, we did not document how many patients did not complete or turn in surveys; however, based on the surveys returned and the number of procedures performed, we feel the number of non-responders was low. Although these limitations prevent us from drawing any conclusions regarding efficacy, the fact that many patients sought multiple SGB procedures suggests patient-perceived benefit.

## Conclusions

Our quality assurance project suggests that in our center the SGB procedure for PTSD is a safe, well-tolerated, and accepted treatment adjunct for the management of symptoms associated with chronic treatment-refractory PTSD. Further studies are indicated to determine the effectiveness, duration of action, and optimal treatment regimen.

## Appendices

Appendix I. Survey Questions:

Dear Patient:  Please take a few minutes to complete this voluntary and anonymous questionnaire.  Your answers will assist us in our on-going effort to improve quality of care.  Thank you.

1.  Which treatment number was this?

a. 1

b. 2

c. 3

d. 4 or more

2.  How uncomfortable was the procedure or you?

a. no discomfort

b. mild discomfort

c. moderate discomfort

d. severe discomfort

3.  How would you rate any side effects you had from this procedure compared to other treatments (medications) you have had.

a. the same

b. less

c. more

4.  Would you be willing to have the procedure again?

a. yes

b. no

5.  Would you recommend the procedure to a friend?

a. yes

b. no

6.  How many procedures would you be willing to have assuming it was an effective treatment?

a.  one more

b.  2-3 more

c.  as many as it takes

7.  How satisfied are you with the process and the education you received prior to and after the procedure?

a. satisfied

b. neutral

c. dissatisfied

8. How can we improve our process?

Overall Satisfied? (Yes, No):

a. yes

b. no

Appendix 2: Interventional Pain Management Service Quality Assurance Indicators

Cardiovascular:

Hemodynamically significant arrhythmia requiring ACLS

Significant hypo or hypertension requiring treatment

Vasovagal even requiring mediation

Respiratory:

Hypoxia

Pneumothorax

Pulmonary aspiration

Neurological:

New CNS/PNS deficit within 48 hours

Paralysis

Increase in patient’s pain requiring hospital admission

Procedural:

Toxic reaction to local anesthetic

Wrong site block

Injection site infection

Drug reaction

Medication errors

Other_____________
